# The Treatment of Ulcers of the Cornea

**Published:** 1898-11-05

**Authors:** 


### THE TREATMENT OF ULCERS OF THE CORNEA.

In a post-graduate lecture on ulcers of the cornea recently delivered at the West London Hospital by Mr. Percy Dunn some interesting details were given as to the various modern methods of treatment which are applicable to these cases. For practical purposes he divides corneal ulcers into (1) traumatic, (2) simple, and (3) complicated, the latter including (*a*) the sloughing, (*b*) the vascular, and (*c*) the infective varieties.

(1) Traumatic: Cocaine should be instilled into the conjunctival sac, and afterwards a warm chinosol solution (1 in 2,000) should be douched over the eye until thorough cleansing has been secured. Atropine drops (grain two to an ounce) may then be instilled, and a pad of chinosol gauze applied and secured with a bandage. The drops may be used night and morning, and the sac washed with chinosol solution at the same time until the eye is well.

(2) Simple: Common among weakly school children. Photophobia variable, sometimes severe, sometimes very slight. If much photophobia be present cocaine and atropine drops may be used twice a day, and the eye at the same time bathed with warm water. A shade should also be worn with a thin layer of cotton wool under it in order to exert some pressure upon the closed upper lid. Tonics should be given, of which tincture of the perchloride of iron with glycerine answers well. If photophobia be not troublesome some atropine drops may alone be used, and their instillation should be continued until all injection of the globe has subsided. Afterwards some dilute yellow ointment may be placed in the conjunctival sac every night at bedtime as a stimulant to the process of repair. School should be given up for the time.

(3) Complicated ulcers: (*a*) Sloughing. Eserine drops of the strength of half a grain to an ounce of sterilised water should be instilled twice or oftener a day. This strength should never be exceeded, but if greater effect is required the frequency of application may be increased. Heat in the form of hot fomentations is absolutely needful. Nothing so well relieves the acute pain which these ulcers often cause. A large pad of chinosol gauze dipped into boiling water and wrung dry should be placed over the lid of the affected eye, and the applications continued for half an hour or more twice or thrice a day. The fomentations in such cases should not contain belladonna—if eserine is being used the drugs would neutralise each other, while if atropine drops are being employed the combination is apt to set up atropine irritation. Good food is necessary, and if the disease occurs during lactation the weaning of the infant becomes an essential part of the treatment.

(*b*) The vascular ulcer is easily amenable to treatment by eserine.

(*c*) The infective ulcer, often called the serpiginous ulcer of the cornea, commonly has a crescentic outline, and begins at the corneal margin. The disease is very destructive, and in the majority of cases calls for vigorous treatment. Much pain, photophobia, and injection of the globe accompany this form of ulceration, and the patients are mostly men of middle age. The case should be treated antiseptically from the first. Cocaine having been instilled, a warm chinosol solution (1 in 2,000) should be allowed to play over the surface of the ulcer, and the conjunctival sac should be thoroughly irrigated. Eserine drops should be instilled, and afterwards finely pulverised iodoform may be dusted over the corneal surface. Lastly, some chinosol ointment should be smeared over the margins of the closed lids and a pad of chinosol gauze and a bandage applied. If still the ulcer continues to spread, the eye having been cocainised, a drop of a solution of fluorescin must be instilled, by which the area of the ulceration will be made to appear of a bright green colour, and to the surface thus delineated the actual cautery must be applied, the margins being especially dealt with. Of course, after such treatment much opacity remains. In regard to the use of eserine it must be remembered that it is seldom if ever necessary to employ a greater strength than half a grain to an ounce, and that for continuous instillation even a less strength may be used. There are certain forms of ulceration of the cornea which atropine fails to benefit, but which, on the other hand, readily yield to eserine. Broadly, it may be said that all sloughing, infective, and vascular ulcers are best treated by eserine, as well as, of course, those situated at the corneal margin at which perforation is threatening.

